# Study on Hydrological Functions of Litter Layers in North China

**DOI:** 10.1371/journal.pone.0070328

**Published:** 2013-07-30

**Authors:** Xiang Li, Jianzhi Niu, Baoyuan Xie

**Affiliations:** Key Laboratory of Soil and Water Conservation and Desertification Combating of Education Ministry, Beijing Forestry University, Beijing, China; University of Kansas, United States of America

## Abstract

Canopy interception, throughfall, stemflow, and runoff have received considerable attention during the study of water balance and hydrological processes in forested ecosystems. Past research has either neglected or underestimated the role of hydrological functions of litter layers, although some studies have considered the impact of various characteristics of rainfall and litter on litter interception. Based on both simulated rainfall and litter conditions in North China, the effect of litter mass, rainfall intensity and litter type on the maximum water storage capacity of litter (*S*) and litter interception storage capacity (*C*) were investigated under five simulated rainfall intensities and four litter masses for two litter types. The results indicated: 1) the *S* values increased linearly with litter mass, and the *S* values of broadleaf litter were on average 2.65 times larger than the *S* values of needle leaf litter; 2) rainfall intensity rather than litter mass determined the maximum interception storage capacity (*C_max_*); *C_max_* increased linearly with increasing rainfall intensity; by contrast, the minimum interception storage capacity (*C_min_*) showed a linear relationship with litter mass, but a poor correlation with rainfall intensity; 3) litter type impacted *C_max_* and *C_min_*; the values of *C_max_* and *C_min_* for broadleaf litter were larger than those of needle leaf litter, which indicated that broadleaf litter could intercepte and store more water than needle leaf litter; 4) a gap existed between *C_max_* and *C_min_*, indicating that litter played a significant role by allowing rainwater to infiltrate or to produce runoff rather than intercepting it and allowing it to evaporate after the rainfall event; 5) *C_min_* was always less than *S* at the same litter mass, which should be considered in future interception predictions. Vegetation and precipitation characteristics played important roles in hydrological characteristics.

## Introduction

In the forest ecosystem, the canopy is regularly recognized as a main re-distributor of rainfall in space [1]. Thus previous studies have put great emphases on canopy interception, throughfall, stemflow and surface runoff under vegetation cover. Meanwhile, the hydrological functions of litter layers appears to be a minor concern [2]–[4]. Litter, which is composed of dead leaves, twigs, branches and other fragmented organic materials, plays a crucial role in hydrological processes by covering the mineral soil and functioning as another main rainfall re-distributor [5].In the short term, litter absorbs the energy of raindrops while preventing soil erosion [6]–[8], helps keep infiltration rates high, and reduces the soil temperature which lowers evaporation rates by insulating the soil surface from the atmosphere [9]–[11]. Over time, decomposed litter plays an important role in changing soil physical properties such as bulk density and pore volume. It also serves as a major source of soil organic matter, strongly influences the structure of soil, and increases soil stability and porosity while increasing the ability of water to infiltrate into the soil [12], [13].

Despite the importance of litter in hydrological processes, the water dynamics of the litter layer are largely underestimated and may even be disregarded in hydrological models [14], mainly because of the technical difficulties in making accurate measurements [14], [15]. Knowing this, several previous studies have been conducted to clarify the role of moisture dynamics of litter especially as it relates to interception loss and evaporation. These studies mainly focused on calculating three parameters, the maximum water storage capacity (*S*), the maximum interception storage capacity (*C_max_*) and the minimum interception storage capacity (*C_min_*). *S* is defined as the amount of water retained by litter when leaf surface is completely wet and thus, litter is fully saturated [5], while Pitman [16], Putuhena and Cordery [17] defined and calculated *C_max_* and *C_min_* for each test run. *C_max_*, is the maximum interception storage capacity of the litter layer, taken as the amount of water retained in the sample immediately before cessation of the rainfall, including water that would later drip away because of gravity; *C_min_*, is the minimum interception storage capacity of the litter layer, taken as the amount of water retained in the sample after drainage had ceased. This amount of water can be removed only by evaporation.

For example, Helvey and Patric [18] reported that the *S* of litter of US eastern hardwoods was 135–170% of the weight of the litter under simulated rainfall, Pitman [16] found that the value of *C_max_*, for bracken litter was 4.84 mm kg^–1^ m^–2^, and the value of *C_min_*, was 1.67 mm kg^–1^ m^–2^ under a rainfall intensity of 120 mm h^−1^. Putuhena and Cordery [17] reported that the *C_min_* was proportional to litter mass per unit area (kg m^−2^) under a rainfall intensity of 34–75 mm h^−1^. Marin *et al*. [19] claimed that the average *C_min_* of the Amazonian forest floor was 1.51 (±) 0.30 mm kg^−1 ^m^−2^. Sato *et al*. [5] obtained similar results under lighter rainfall intensities and compared the *S*, *C_max_*, and *C_min_* of two litter types. Their results suggested that, broadleaf litter stored more water than needle leaf litter. Guevara-Escobar *et al*. [20] investigated the effect of rainfall intensity on *C_min_*; their results indicated that *C_min_* was not influenced by rainfall intensity. This finding differed from those of Putuhena and Cordery [17] and Sato *et al*. [5]. The discrepancies found in former studies indicated that further research is needed to clarify the hydrological functions of the litter layers. For example, previous authors made their conclusions based on extremely high rainfall intensities (e.g. [16], [17]). Little information is available about light-intensity rainfalls. Further, few experiments have been conducted on the effect of litter characteristics such as litter mass and litter type on *S, C_max_* and *C_min_*. Only Sato *et al*. [5] mentioned litter types, but they chose only one species for each type in Japan, and so their results cannot adequately explain the phenomenon. Furthermore, while former studies have focused on interception loss or water erosion under mulch covers like leaf litter, grass, rocks or pebbles in a semiarid region of West China or South China (e.g. [21]–[24]), few have been carried out in North China.

So our study aimed to: 1) discover *S* of litter layers for two litter types in North China and their relationship with litter mass; 2) determine the effect of litter mass, rainfall intensity and litter type on *C_max_* and *C_min_*; 3) illustrate the hydrological role of litter layers by observing the variation of *C_max_* and *C_min_* values during and after a rainfall event, and explore the *C_max_*–*C_min_* relationship; 4) compare the values of *S* and *C_min_*, and evaluate their utility to predict or calculate interception loss.

## Materials and Methods

### Ethics Statement

The experimental site, Jiu Feng National Forestry Park is managed by the Forestry Committee of Beijing Forestry University and is available for teaching and research of the university. This field study did not involve any endangered or protected species, and all the four tress species we selected were common species in North China.

### Study Site

The experimental site, Jiu Feng National Forest Park, is located in northwestern Beijing, China (116°28′E, 39°34′N) with a warmer temperate climate with hot wet summers and cold dry winters. The mean daily temperatures range between 23°C and 28°C from May to mid-October and –5°C and 14.7°C in winter with a mean annual temperature of 11.6°C. Mean annual precipitation is 630 mm, most of which falls as rain between June and September. Very intense and erosive rainfall events usually occur in autumn after the summer drought period. The dominant tree species include *Pinus tabulaeformis*, *Quercus variabilis*, and *Platycladus orientalis*, all of which were planted from the 1950s to 1960s. The poorly developed soil contains little organic matter. Soil types change with elevation with cinnamon soils at 70−900 m and brown soils above 900 m. The general slope averages 10% with a northeast aspect.

### Litter

Based on the stand characteristics, litter physical characteristics and availability (Table 1), four dominant types of litter were collected and tested: *Quercus variabilis* and *Acer truncatum* litter represented broadleaf litter and *Pinus tabulaeformis* and *Platycladus orientalis* litter represented needle leaf litter. In four 25×15 m^2^ experimental stands (one for each litter species), we investigated the unique distribution of the litter layer using 10 randomly selected 1×1 m^2^ plots which were installed temporarily by positioning an aluminum sheet above the ground to estimate the litter mass. Because it was very difficult to overcome the influence of wind and mass variations from plot to plot, we collected the litter in the plots by hand. Then the litter was placed in plastic bags and brought back to laboratory for analysis. After collection, litter was allowed to dry naturally in the laboratory. We also determined the litter lengths and widths for each litter species. For most needle leaves, since the twigs and needle leaves fell together and were difficult to separate, we treated them as whole units for calculations and weighing. In the weighing process, we removed the decomposed litter. The undecomposed litter was used for further tests, for two main reasons; first, it was the dominant component of the litter (approx. 85%) and was able to be separated easily from the whole sample to test in the sample tray; second, the undecomposed litter was located in the upper layer and it was positioned to intercept and store the raindrops directly. Thus, we believe that the undecomposed litter should play a more significant role in the rainfall-interception process because of its physical characteristics; that is, the larger leaf area and more abundant intact surface trichomes compared with the decomposed litter. Ultimately we found the broadleaf and needle litter masses varied from 0.33 to 1.24 kg m^–2^ and 0.18 to 0.77 kg m^–2^, respectively.

**Table 1:** Forest stands and litter characteristics.

Species	Plot area (m^2^)	Density (trees ha^−1^)	DBH 1 (cm)	Height (m)	Litter length (cm)	Litter width (cm)
*Q. variabilis*	25×15	1225	11.2	9.8	11.3–13.7	3.5–4.3
*A. truncatum*	25×15	2371	10.5	7.8	6–10	5–6.2
*P. tabulaeformis*	25×15	1748	7.6	5.2	9.5–12.5	3.3–5.1^a^
*P. orientalis*	25×15	3002	5.8	3.4	7.2–10.1	2.4–4.1^b^

1 DBH means tree diameter at breast height.

a,b width data for needle-leaf litter included twigs and needles.

### Rainfall Simulator

The artificial rainfall simulator was developed jointly by Beijing Normal University and Beijing Jiaotong University in 2006 [25], [26]. The simulator was equipped with a Veejet 80150 sprinkler (Spraying Systems Co., Wheaton, IL, USA). Three nozzles on rotary axle mounts were spaced 1.1 m apart. The simulator oscillates at a height of 4.5 m with varying speeds, and sprays an area 2.2 m long×1.5 m wide with various rainfall intensities. This arrangement was designed to allow raindrops to reach terminal velocity, simulating a stable rainfall event with more than 0.8 rainfall uniformity and raindrops 2.3±0.3 mm in diameter.

### Interception Device

**Figure 1:**
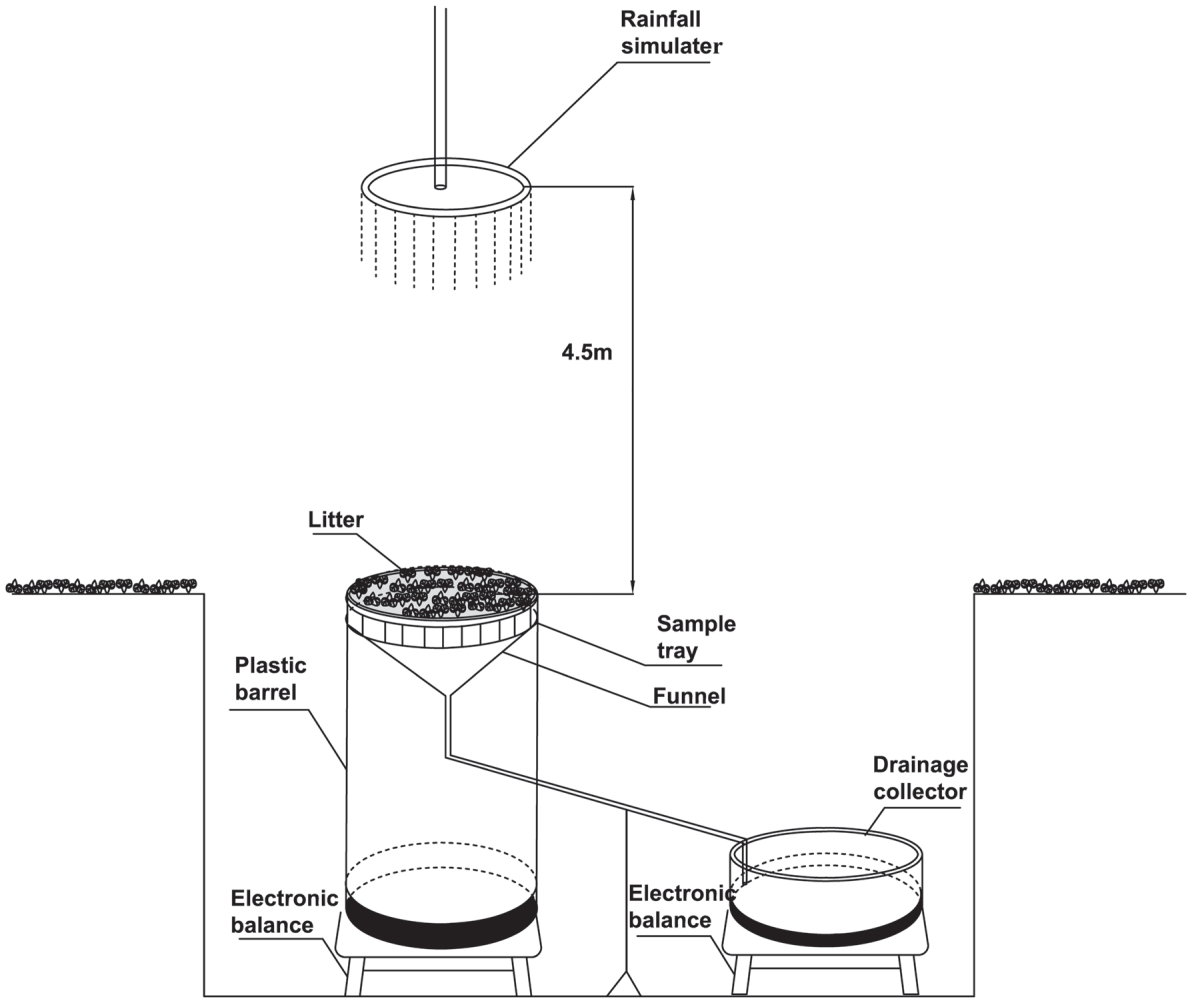
Schenmatic representation of interception device.

The interception device consisted of a sample tray, plastic barrel, funnel, drainage collector and an electronic balance (Fig. 1). The 48cm-diameter circular sample tray was located above a plastic barrel. Several 3 mm diameter strands constituted a 2×2 cm^2^ mesh. When rainfall began, water drained from the litter and flowed through the funnel and the tube to the drainage collector, where it was, weighed by an electronic balance every minute.

### Measurements

#### Maximum water storage capacity (*S*)

*S* is reached only when litter is fully saturated. It is especially useful as a significant indicator of interception loss in evaluating the water dynamics of litter. We tested *S* using litter masses of 0.3, 0.5, 0.8, and 1.0 kg m^–2^ for each species. After preparing each sample on a tray, we soaked it in water for 24 h to saturate the sample completely. Then, the sample was lifted from the water and allowed to air dry and drain for about 30 min, before being weighed with an electronic balance. The difference in the litter mass before and after the soaking procedure was taken as *S*. Each sample type for each litter mass was replicated three times.

#### Interception storage capacity (*C*)

*C* may be the most significant indicator of interception loss when evaluating the hydrological functions of litter layers. For each species, values of *C_max_* and *C_min_* were calculated under five rainfall intensities, 6.8, 13.5, 37.7, 74.4, and 115 mm h^–1^, and four litter masses, 0.3, 0.5, 0.8, and 1.0 kg m^–2^, for durations of 60 min to measure the effect of litter mass, litter type and rainfall intensity on *C_max_* and *C_min_*. Though 60-minute rainfall under this range of rainfall intensity may not be sufficient to saturate the litters to obtain the ‘maximum’ values of *C_max_* or *C_min_*, these ranges of rainfall intensity, rainfall duration and litter mass were considered to represent the natural conditions of the experimental site, because natural rainfall at the site is relatively stable in 60 min, but can fluctuate severely over long periods, especially since 1984 [27], [28]. However, it is worth noting that a 60-minute rainfall under this range of rainfall intensity may not be sufficient to saturate the litters to obtain the ‘maximum’ values of *C_max_* or *C_min_*. For each test run, drainage water was collected and recorded every minute. Therefore, *C* was measured as precipitation minus drainage. When the artificial rainfall ceased, *C_max_* was equal to the accumulation of *C* of every min; after the drainage ceased, *C_min_* was calculated by the mass difference of the litter samples before and after the rainfall event.

### Statistical Analysis

*S*, *C_max_* and *C_min_* were analyzed separately using four litter masses and five rainfall intensities for each species. The relationships between litter mass and *S*, litter mass and *C_min_*, rainfall intensity and *C_max_* were tested using the linear fitting function in SPSS. Analysis of variance (ANOVA) was used to test differences using the Listwise Statistics Dependent test at *p*<0.01. All statistical analyses were performed using SPSS software.

## Results and Discussion

We investigated the hydrological functions of litter layers under four litter masses and five rainfall intensities for four litter species. We analyzed the relationship between *S* and litter mass, and litter type. When analyzing *C_max_* and *C_min_*, Putuhena and Cordery [17] and Sato *et al*. [5] claimed that *C_min_* was more important than *C_max_* because gravitational water readily drains within about 30 min after the cessation of rainfall. However, *C_max_* represents the amount of water that dampens the soil during intense rainfall [20], [30], creating a buffer layer in the litter which protects the soil from splashing. Therefore, the gap between values of *C_max_* and *C_min_* may be meaningful to fully clarify the hydrological functions of litter layers. In this study, we discussed the effect of litter mass, litter type and rainfall intensity on both *C_max_* and *C_min_*. In addition, we focused on the huge gap between the values of *C_max_* and *C_min_*, and compared the values of *S* and *C_min_*.

### Maximum Water Storage Capacity (*S*)

**Figure 2:**
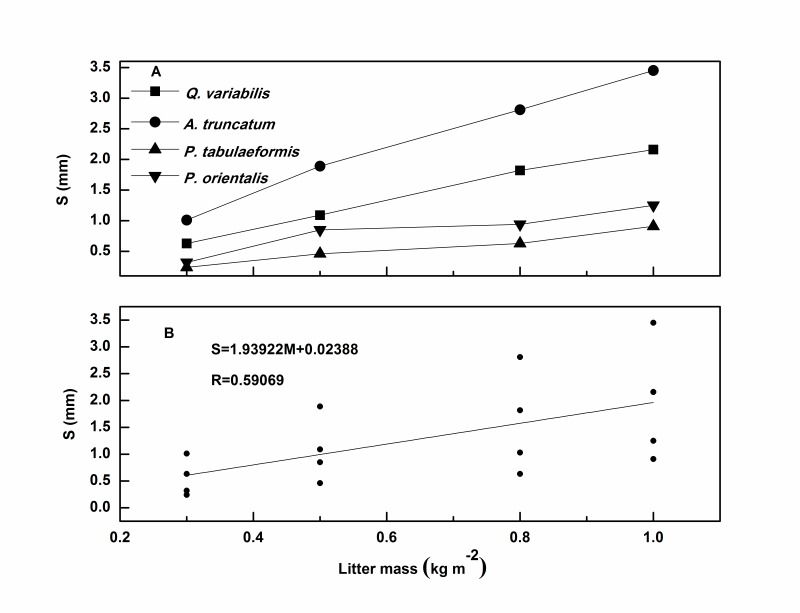
Maximum water storage capacities (*S*) of four litter species and its relationship to litter mass.

We determined *S* values using different litter masses for each sample (Fig. 2A). Generally, the values of *S* ranged from 0.24 mm to 3.45 mm when the litter mass increased from 0.3 kg m^–2^ to 1.0 kg m^–2^. Because of its greater leaf area, broadleaf litter could absorb more water than the same mass of needle leaf litter. This gap increased rapidly with an increase in litter mass. The *S* values of broadleaf litter were, on average, 2.65 times larger than the *S* values of needle leaf litter. These results implied that litter type played an important role in determining *S*.

This conclusion differed from the findings of Sato *et al.* [5]. In their study, they argued that *S* was relatively constant regardless of litter types and shapes; furthermore, their reported *S* values were within the range reported in several previous studies, e.g., Pitman [16], Putuhena and Cordery [17], Tobon-Marin *et al*. [19], Crockford and Richardson [29],. However, all of these studies were carried out under rainfall, so their experimental conditions were quite different from those of Sato *et al*. [5] and those of the present study. Specifically, as they stated, the values they obtained were measures of *C_min_*, not *S*, because the litter was not completely saturated under the simulated rainfall as was done in previous studies.

Similar to the findings of Sato *et al*. [5], we found *S* showed a strong linear relationship with litter mass, not only for each species but also when data for all species were combined together (Fig. 2B). The results showed that litter mass has a strong influence on *S* regardless of litter type.

### Interception Storage Capacity

#### Effect of litter mass on C_max_ and C_min_

**Figure 3:**
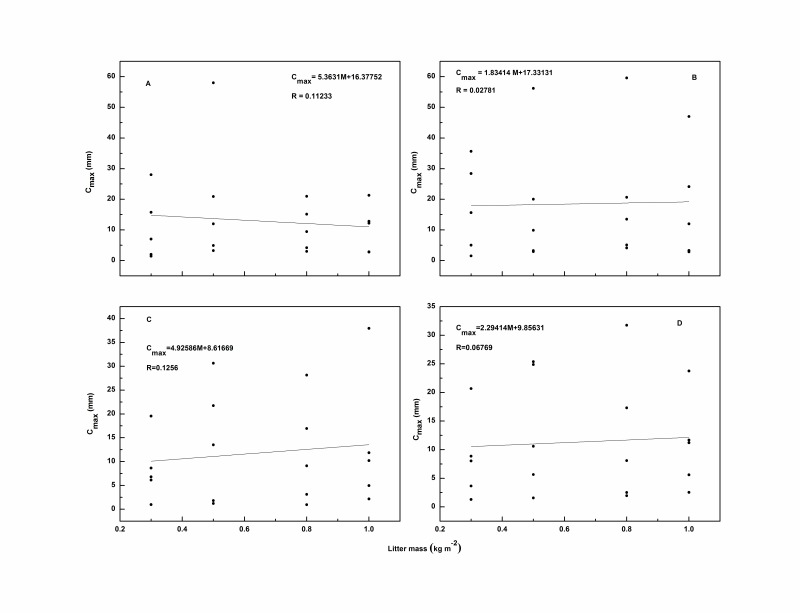
Relationships between litter mass and *C_max_* of *Q.variabilis* (A), *A*. *truncatum* (B), *P. tabulaeformis* (C), *P. orientalis* (D). These values were obtained using five rainfall intensities. To clarify the relationship between litter mass and *C_max_* (or *C_min_*), rainfall intensities are not shown in Figure 3–5.

There were no obvious linear relationships was observed between litter mass (kg m^–2^) and *C_max_* for each species (Fig. 3), different from the findings of Putuhena and Cordery [17]. *C_max_* increased as litter mass increased from 0.3 to 0.5 kg m^–2^ for the needle leaf species but decreased when the litter mass increased from 0.8 to 1.0 kg m^–2^. However, with increasing litter mass, *C_max_* tended to be relatively constant for *Q.variabilis* and fluctuate greatly for *A.truncatum*.

The discrepancy between our findings and those of Putuhena and Cordery [17] was probably because of the different litter masses used. They tested samples using masses in the range of 0.5 to 6 kg m^–2^, compared with the range of 0.3 to 1 kg m^–2^ we chose. Aside from the litter mass, different materials may also influence the results; they collected an undisturbed sample for two species, and found the boundary between mineral soil and litter was not sharp especially for the *Eucalyptus* litter. By comparison, the litter we used was relatively undecomposed for all four species. The variations in the collected data may also be caused by the contact angle between the upper leaves and the lower leaves. During a rainfall event, with the continual impact of raindrops, the contact angle between leaves and the surface sloped and created some macro pores, which potentially provided a preferential flow channel for the rainwater, especially in needle leaf litter. Thus, a proportion of rainwater may have drained from the litter layer during or after the rainfall event, largely depending on the variations in the litter sample structure such as macro pores and surface pits. The phenomenon implied that the dominant force influencing *C_max_* was not capillary force but gravity and cohesion forces; this was consistent with the findings of Guevara-Escobar *et al*. [20]. However, the differences between the findings of Putuhena and Cordery [17] and those of the present study indicated the relationship between litter mass and *C_max_* deserves further research. The results also implied that litter morphological characteristics played a particular role that needs to be considered and studied further.

**Figure 4:**
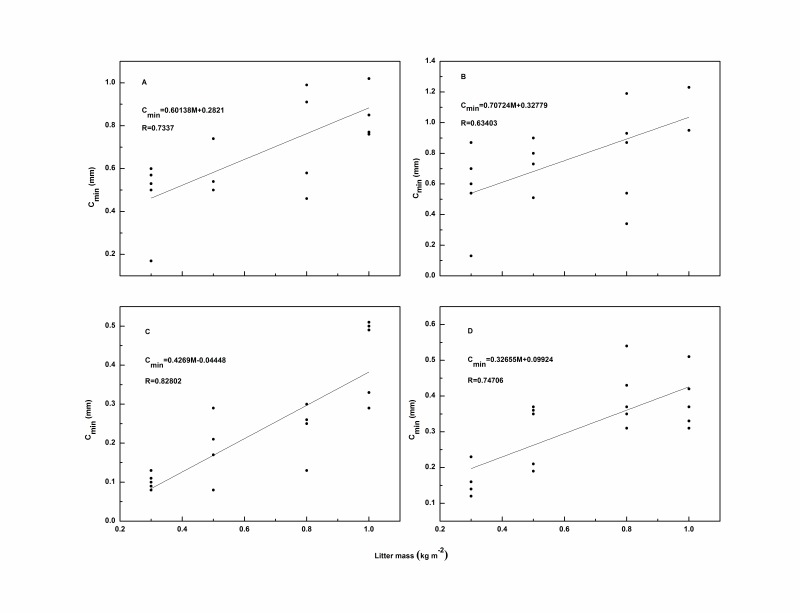
Relationships between litter mass and *C_min_* for *Q. variabilis* (A), *A*. *truncatum* (B), *P. tabulaeformis* (C), and *P. orientalis* (D).

**Figure 5:**
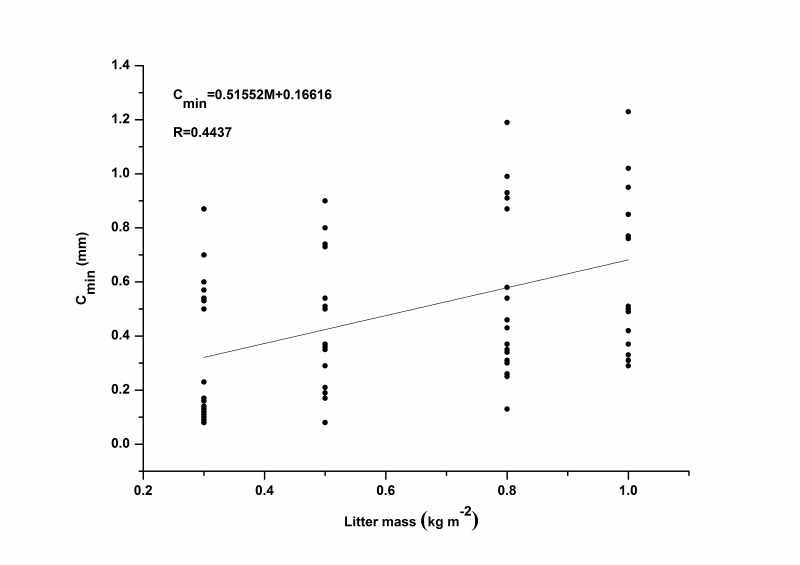
Relationships between litter mass and *C_min_* for combined data from all four species.

Compared with *C_max_*, a linear relationship was found between litter mass and *C_min_* with the correlation coefficient (*R*) greater than 0.6 for each species (Fig. 4). Evidently, *C_min_* increased with increasing litter mass regardless of the rainfall intensity. This may be because after the cessation of simulated rainfall, the intercepted rainwater continued to drain through every layer of the sample, drip by drip. However, when the litter mass increased, more physical barriers in the litter layer emerged, especially in broadleaf litter. These barriers intercepted and stored more rainwater, preventing it from draining, similar to the adhesion of water held by the surface of each piece of litter [5]. For the needle leaf litter, some rainwater passed directly through the macro pores of the litter as raindrops were intercepted and splashed during the rainfall event. Because the remaining raindrops fragmented into smaller droplets, they were readily absorbed by the underlying needle leaves and twigs. These results were consistent with the conclusions of Putuhena and Cordery [17], Sato *et al*. [5]and Guevara-Escobar *et al*. [20]. However, the relationship between litter mass and *C_min_* was not completely clear regardless of litter type. The linear correlation coefficient (*R*) decreased to 0.4437 (Fig. 5), a value that was much smaller than that for each individual species (Fig. 4), revealing an obvious difference in *C_min_* between broadleaf litter and needle leaf litter and indicating that not only litter mass but also litter type played a vital role in determining of *C_min_*.

#### Effect of rainfall intensity on C_max_ and C_min_

**Figure 6:**
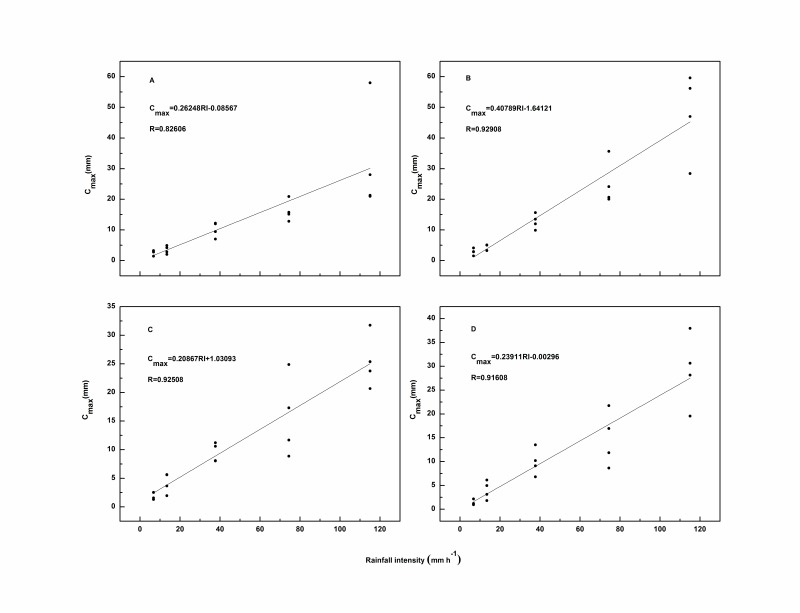
Relationships between rainfall intensity and maximum interception storage capacity (*C_max_*) for *Q.variabilis* (A), *A*. *truncatum* (B), *P. tabulaeformis* (C), *P. orientalis* (D) These values were obtained under four litter masses. To clarify the relationship between rainfall intensity and *C_max_* (or *C_min_*), litter masses are not shown in Figures 6–7.

**Figure 7:**
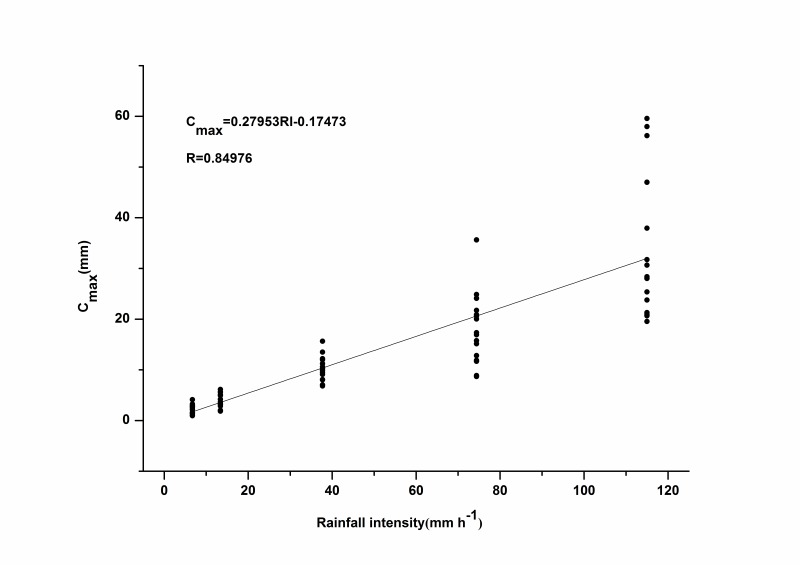
Relationship between rainfall intensity and *C_max_* combined data from all four species.

Rainfall intensity is thought to be a key factor influencing the interception storage capacity [5]. As expected, there was a strong linear relationship between rainfall intensity and *C_max_*, with *R* values greater than 0.8 (*p*<0.05) for each species (Fig. 6). Furthermore, *C_max_* generally increased with increasing rainfall intensity regardless of litter mass and species (Fig. 7). This phenomenon was observed even under extremely high rainfall intensities (115 mm h^–1^), although several researchers drew different conclusions. Putuhena and Cordery [17] believed a clear relationship did not exist between *C_max_* and rainfall intensity. They observed only slightly higher *C_max_* values with higher rainfall intensities, and calculated *C_max_* for the rainfall intensities of 48 and 68 mm h^–1^. Sato *et al*. [5] claimed that *C_max_* increased with increasing rainfall intensity in realistic rainfall conditions (less than 50 mm h^–1^). Guevara-Escobar *et al*. [20] reported that *C_max_* increased with increasing rainfall intensity in the case of poplar leaves under rainfall intensities of 9.8, 30.2, 40.4, and 70.9 mm h^–1^. These differences may be caused by differences among the various simulated rainfall conditions studied. Putuhena and Cordery [17] studied *C_max_* until the drainage rate stabilized under rainfall intensities of 34 to 75 mm h^–1^, and the rainfall duration was 30 to 60 minutes. By comparison, Sato *et al*. [5] applied three rainfall intensities, 5, 10, and 50 mm h^–1^ for durations of 300 min to obtain *C_max_*. Guevara-Escobar *et al*. [20] tested samples under 60 min simulated rainfall. These different methods resulted from various precipitations for each sample, so some litter samples reached the ‘maximum’ C*_max_* and some did not. Another factor influencing rainfall interception may be the different materials used. In our study, the broadleaf litter was composed of hairy leaves with trichomes, which strongly influenced surface tension and contact angle [20]. Leaf physical characteristics may affect the ability of litter to store additional rainwater during a rainfall event.

**Figure 8:**
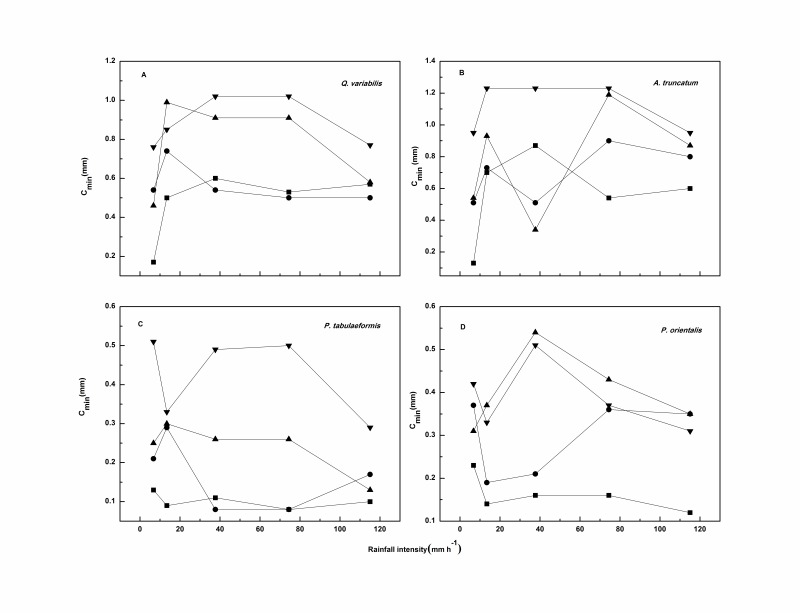
Relationships between rainfall intensity and *C_min_* for each species. Squares, circles, downward triangles, and upward triangles represent litter masses of 0.3, 0.5, 0.8, and 1.0 kg m^–2^, respectively.

The effect of rainfall intensity on *C_min_* was not clear and no linear relationship between the two was observed (Fig. 8). The *C_min_* values of broadleaf litter tended to increase rapidly when rainfall intensity increased from 6.7 to 13.5 mm h^–1^; the increment ranged from 0.09 to 0.57 mm. It remained constant or decreased at moderate rainfall intensities but decreased greatly at the extremely high rainfall intensity of 115 mm h^–1^; the decrement averaged 0.15 mm. By contrast, the needle leaf litter showed a trend to decrease, increase, and then markedly decrease when the rainfall intensity increased gradually from 6.7 to 115 mm h^–1^.

The phenomenon can be explained as stated above. That is, the raindrop diameter increased with increasing rainfall intensity, allowing more raindrop kinetic energy to accumulate before the drops hit the surface of the litter sample. The leaf physical characteristics should be taken into consideration [10], [20], [30]. The leaf contact angle formed a steeper gradient with increasing rainfall intensity, allowing for the creation of additional macro pores and the formation of a preferential flow channel. Therefore, although the leaf stored a large proportion of rainwater in each layer during more intense rainfall, the surface tension was overcome by the increased gravity of the stored rainwater. This resulted in a large volume of rainwater draining away after the rainfall, as *C_min_* was measured when the drainage ceased. While less rainwater was stored at lower rainfall intensities (6.7, 13.5 mm h^–1^), surface tension was difficult to overcome; therefore a smaller proportion of water drained away, resulting in a smaller value for *C_min_* at 115 mm h^–1^ compared with that determined at 6.7 mm h^–1^.

Concerning the impact of rainfall intensity on *C_min_*, previous researchers drew various conclusions based on the different materials and rainfall conditions studied. Putuhena and Cordery [17] reported that *C_min_* was rather constant with rainfall rates of 34 to 75 mm h^–1^. Similar to C*_max_*, Sato *et al*. [5] found *C_min_* increased with increasing rainfall intensity (less than 50 mm h^–1^). Guevara-Escobar *et al*. [20] found that *C_min_* remained fairly constant with respect to rainfall intensity for poplar leaves and fresh grass, and decreased with increasing rainfall intensity for woodchips.

#### Effect of litter type on C_max_ and C_min_

Litter type was supposed to be a dominant factor in determining interception storage capacity [5], [20]. Based on the above analysis, we found the *C_max_* values ranged from 1.43 to 59.57 mm and averaged 15.71 mm for broadleaf litter (*Q*. *variabilis* and *A*. *truncatum*, respectively). For needle leaf litter from *P. tabulaeformis* and *P. orientalis*, the *C_max_* values ranged from 0.99 to 31.75 mm and averaged 11.58 mm. Similarly, the *C_min_* values ranged from 0.13 to 1.23 mm, and averaged 0.73 mm for broadleaf litter. For needle leaf litter, the *C_min_* values ranged from 0.08 to 0.54 mm and averaged only 0.27 mm. These results indicated that broadleaf litter could intercept and store more rainwater than needle leaf litter because the larger flat leaf area of broadleaf litter was able to control the spread of rainwater. Also litter porosity and surface tension played a crucial role in determining *C_max_* and *C_min_*. The conclusion was consistent with the findings of Sato *et al*. [5] and Guevara-Escobar *et al*. [20]. The gap between broadleaf litter and needle leaf litter in *C_max_* and *C_min_* became wider because of the differences in materials and rainfall conditions.

### Relationship between *C_max_* and *C_min_*

When rainfall ceased, intercepted rainwater continued to drain so that *C_min_* was reached in nearly 30 min. Rainwater continued to infiltrate into the soil or form runoff, therefore, the difference between *C_max_* and *C_min_* is an important indicator that can be used to evaluate the effect of the litter layer in protecting the soil from erosion in the rainfall-interception process. In our study, we obtained extremely high values of *C_max_* compared with those in former studies. Generally, the *C_max_* values in the present study ranged from 0.99 mm to 59.57 mm, while those in previous studies ranged from 1 to 6 mm. This huge difference was caused by not only the experimental materials but also by the different methods used to simulate rainfall and modify rainfall intensities. However, there were smaller differences among *C_min_* values, which ranged 0.08 to 1.23 mm in the present study, compared with 1.67 mm reported by Pitman [16], 0.96 and 1.12 mm reported by Putuhena and Cordery [17], 0.27 to 3.05 mm reported by Sato *et al*. [5], and 0.4 to 0.7 mm reported by Guevara-Escobar *et al*. [20].

The results also revealed that for all the species, 27.58% of total rainwater (∑*C_max_*/∑precipitation) was intercepted during the rainfall but continued to drip after the rainfall, so that only 1.01% of total rainwater (∑*C_min_*/∑precipitation) was stored by litter and successively evaporated over a few hours. The large difference between *C_max_* and *C_min_* in our study indicated the impact of the litter layer on rainwater. The litter layers transformed the intercepted rainwater causing it to infiltrate into the soil or produce runoff rather than intercepting it and allowing it to evaporate after the rainfall event.

### Comparison between *S* and *C_min_*

**Figure 9:**
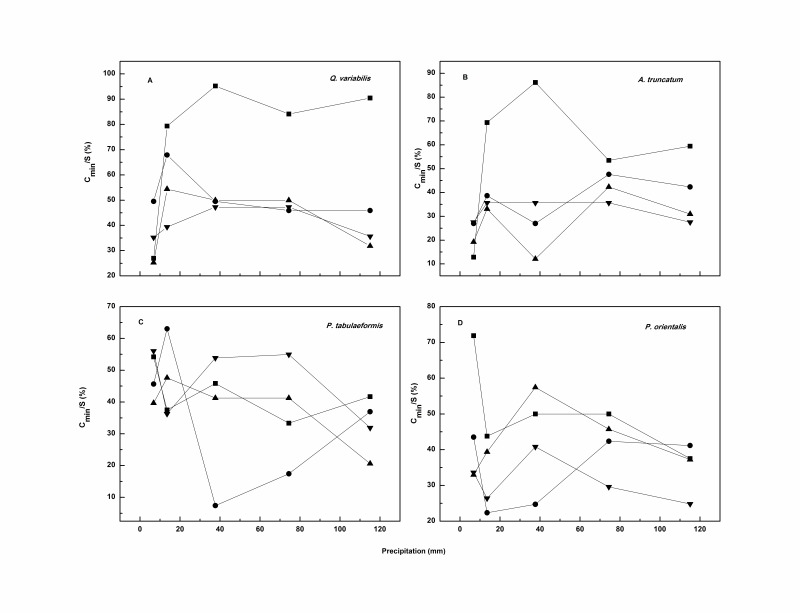
*C_min_* accounts for percentage of *S* with precipitation and litter mass. Squares, circles, downward triangles, and upward triangles represent litter masses of 0.3, 0.5, 0.8, and 1.0 kg m^–2^, respectively.

Given the analysis above, both *S* and *C_min_* showed a linear relation with litter mass. The comparison between *S* and *C_min_* for each species under the litter mass 0.3 to 1 kg m^−2^ revealed the *C_min_*/*S* of *Q*. *variabilis* ranged from 25.27% to 95.24%, and averaged 52.55%. For *P. tabulaeformis*, it ranged from 17.39% to 63.04%, and averaged 40.82%. A similar trend was found for the other two species. Apparently, the value of *C_min_* was always smaller than *S* with the same litter mass regardless of the species (Fig. 9). This indicated that the litter layer was not saturated under rainfall conditions. The results were not in agreement with Sato *et al*. [5]. They argued that some relative values of *C_min_*/*S* exceeded 100% because the upper part of the litter layer could catch rainwater in its surface pits. The discrepancy was probably caused by two factors, the rainfall conditions and the structure of the materials. Sato *et al*. [5] simulated rainfall for 300 min to fully saturate the sample and obtained the ‘maximum’ value for *C_min_*. In contrast, we tested the sample for 60 min of rainfall. As noted above, the litter was relatively undecomposed and the leaves were not as curled like those in the study of Sato *et al.* [5], so that few pits were observed in the broadleaf litter surfaces after rainfall.

As a result, the values of *C_min_* were always smaller than those of *S*. So the *S* values were probably able to be used as a parameter in hydrological models or even to predict the interception and evaporation loss under various circumstances.

### Conclusion and Suggestions

The experiment was conducted to investigate the hydrological functions of the litter layer under four tree species, with broadleaf litter represented by *Q*.*variabilis* and *A*. *truncatum* and needle leaf litter represented by *P. tabulaeformis* and *P. orientalis*. The effect of litter mass, litter type, and rainfall intensity on *S*, *C_max_* and *C_min_* were tested under five rainfall intensities and four types of litter mass.

We found a linear relationship between litter mass and *S*, not only for each species but for combined data from the four species. The value of *S* for broadleaf litter was, on average, 2.65 times greater than that for needle leaf litter. A strong linear relationship was observed between rainfall intensity and *C_max_* for each species individually and for the four species combined. This revealed that *C_max_* increased with increasing rainfall intensity. By comparison, *C_min_* was determined by litter mass rather than rainfall intensity and *C_min_* increased with increasing litter mass. However, there was a large gap between *C_max_* and *C_min_*, indicating a large amount of rainwater drained after the rainfall event. Thus, litter played a significant role in transforming the rainwater as it infiltrated into the soil or produced runoff, rather than intercepting it and allowing it to evaporate after the rainfall event. Our results showed that broadleaf litter intercepted and stored more rainwater than did needle leaf litter because of the differences in leaf area, macro pore size and contact angle. Last, and importantly, the values of *C_min_* were always smaller than those of *S*, which emphasized the importance of predicting the interception storage capacity and evaporation in future studies. These results showed that both vegetation characteristics and precipitation characteristics played important roles in hydrological functions of the litter layers.

In the future, variations in litter layer thickness and litter porosity should be considered when analyzing the effects of litter structure not only on litter interception, but also on the movement of water at the litter surface, the movement of water inside litter, and even at the interface between the litter and soil layers. Hydrological models could be created and tested to fully clarify the effects of the litter layer on hydrological processes and to improve the efficiency of water resource management in forest ecosystems.
